# Relationships make research-and researchers – whole

**DOI:** 10.1590/1980-220X-REEUSP-2023-E001en

**Published:** 2023-08-11

**Authors:** Kim Mooney-Doyle, Janet A. Deatrick

**Affiliations:** 1 University of Maryland, School of Nursing, Baltimore, MD, USA.; 2 University of Pennsylvania, Philadelphia, PA, USA.

This past March, we had the great honor to be invited to the University of Sao Paulo School of Nursing as Visiting Professors. We had the privilege to join with the Interdisciplinary Nucleus for Loss and Grief Research (NIPPEL) for their conference presentations and collaborative activities, such as the “Global Considerations in Family Ethics Conference” presented in partnership with the International Family Nursing Association (IFNA). The Visiting Professorship was grounded in a long-standing relationship that began more than 15 years ago among Drs. Regina Szylit, Janet Deatrick (University of Pennsylvania), and Debra Wiegand ([decreased]University of Maryland) and expanded in 2015 to include Drs. Maiara dos Santos (University of São Paulo School of Nursing) and Kim Mooney-Doyle (University of Pennsylvania and presently University of Maryland) and others with whom we collaborate. Through the growth of our relationships over the years, we have grown as scholars and as professionals. This trip solidified our belief about the role of relationships in research and provided evidence for our belief that relationships will be foundational for the evolution of nursing science and nurse scientists. In this editorial, we offer for your consideration the following assertion: relationships make research and researchers whole.

## Relationships are a Way to Steward the Profession

What is the significance of relationships in research? Relationships connect individual researchers with team members, participants, institutions, community, organizations. Good and healthy relationships that are sensitive to and appreciative of where people come from and where they want to go offer protection and nurturance for all involved. As researchers who may be in a role of primary investigator, community partner, mentor, educator, and collaborator, these relationships offer us the opportunity to be good stewards of the integrity, intellectual curiosity, passion, and stories our collaborators bring to our interactions. They also help us to understand what is at stake for others as they embark on research relationships; sharing our inquiry, struggles, fears, and desire to improve the human condition can illuminate our vulnerabilities and our strengths. Good relationships allow us to be good stewards. Such stewardship sets up all involved for success because they denote good faith, respect, and positive regard^(1,2,3)^.

## Relationships are a Source of Inspiration

Relationships remind us why we chose this path and are the nexus of learning communities^(4)^. Through relationships and community, researchers learn and grow and provide a mechanism for teaching and mentoring others^(5)^. The questions that plague us in clinical work and keep us up at night are further clarified through discussions and projects curated within the context of relationships and community. Our spirit of inquiry and desire to discover “the what” and “the how” are grown and negotiated within our social networks. The thrill of finding something new or articulating a new idea is best experienced in the context of relationships with research participants, research partners, collaborators, and students. In the case of less fortunate relationships, our inquiring spirits can be weeded and uprooted to help us discern better practices for the future.

## Relationships are Invisible Strings that Connect us to One Another

Relationships link us with research participants and partners, students, and collaborators even after a project is complete or training has ended. They are the key ingredient that instigate change and action to advocate for a community, follow a line of inquiry, start a new project, or move from trainee or mentee to mentor. Relationships are also the string that bind us together across time, space, and place and through which we are changed. Because we believe in the power of relationships in research and advancing our profession, we offer tips for cultivating and honoring the relationships in your research life below.

When applied to organizations and systems, parallel processes are how the dynamics of one system are picked up and reenacted by another system. Thus, in the case of our long-standing collaboration, we understand that our positive relationships impact our relationships with other researchers, research participants and partners, students, and collaborators. While we are all starting from different, complementary places, we are all traveling together toward one destination- family health and improving the human condition. Here are tips for growing international research collaborations and their implications for researchers, students, schools, and the science.

*Tips for Growing Impactful International Research Collaborations*

1. Keep working on it
  While pressures exist to address other professional priorities, keep in mind that you have to keep tending to these relationships in terms of responding to requests and being open to new projects and ways of understanding phenomenon (in our case family science). Otherwise, you will not even see opportunities or make them work. For example, we ventured as a team into collaboratively creating an innovative research lens to examine a data set on families who experienced the loss of a child. This led to a radically new understandings about provider- family relationships^(6)^. While the process of analysis was laborious, we persevered even in the midst of personal challenges; we kept each other engaged and motivated.
  The Visiting Professorship itself is another example of “working it.” When we understood that we would be traveling to Brazil we had only a few weeks to prepare for the trip, including making personal and professional arrangements. Until that time, it was possible, but Drs. Szylit and dos Santos had to work out many details before they could issue the final invitation. In addition, Drs. Szylit, dos Santos, and Mooney-Doyle were busy integrating the 2^nd^ International NIPPEL Meeting (March 6, 2023) and the International Family Nursing (IFNA) Global Considerations in Family Ethics Conference (March 7, 2023). Our shared history kept everyone optimistic and moving forward, even in the midst of uncertainty. In the end, both conferences and the Visiting Professorship were great successes. Forty participants participated online for the NIPPEL Conference and 142 participated online and in person for the IFNA Conference. Drs. Deatrick and Mooney-Doyle participated in the discussion and critique of thirteen student projects with students at University of Sao Paulo School of Nursing, ranging from undergraduate to graduate students of Drs. Szylit and dos Santos.
2. Partners are essential.
  While we all aspire toward our goals, we need resources to accomplish our goals. By partnering and developing relationships with people, organizations, and communities we have learned to share resources and keep our dreams alive. For example, we invite students’ involvement in all of our work, sharing their training and critique. This process not only invigorates our work but also that of the students as we take them to the clinics, schools, communities, and organizations which make up the social fabric of people’s lives. Some students are involved who want to create programs of research that are related to our research; other students are involved who want to integrate the training into different areas of research. These experiences provide opportunities to invest your time and energy that will ultimately GIVE resources back. While researchers can be competitive about resources/scarcity in their own workplaces, relationship-oriented collaborations provide the potential to level the playing field and share resources which can pay tremendous personal and professional dividends, like our experiences of being Visiting Professors.
3. Leadership and following are both important.
  Everyone on the team can and should assume both leadership roles and follower roles. Leadership can be assumed by researchers, research participants and partners, students, and collaborators research partners. At those times, we must provide the space for them to do so and be a follower to support and co-create with them. We watched this in action at the 2^nd^ International NIPPEL Meeting when NIPPEL student members worked hard and provided leadership while Drs. Szylit and Dos Santos validated their leadership to help create tomorrow’s leaders in palliative care.
4. Have a plan about where to meet potential collaborators.
  Where do you meet potential collaborators? We have found it best to focus on organizations that have an international mission and/or vision in our areas of expertise like IFNA, an organization with nearly four hundred members who represent some thirty-three countries. Organizations like NIPPEL who are closer to home are also vital. You must be willing to put in the work and stretch yourself throughout the process, e.g., using an interpretive vs. descriptive lens during an analysis; being flexible and trusting other’s expertise. Focus on growing relationships- based topics that are of general, shared, and practical interest to investigate, like the emotional impact of the stories we hear in research interviews and strategies to protect researchers. Starting with more practical problems taps into the shared experience of researchers and provides a place to grow substantive work.
5. Enjoy each other.
  We find that we talk less about the experiences of being in the trenches together and doing the work and more about the friendship that anchors us to the work when we celebrate our successes and acknowledge our challenges. During the Visiting Professorship we had opportunities to rejoice with others and experience how others mark special occasions. Care was taken at each opportunity to respect cultural practices, for instance, at a weekend Brazilian family BBQ a vegetarian option was provided for Dr. Mooney-Doyle and each Brazilian dish was explained and displayed with pride.
6. Keep grounded and stay on course.
  As we go through this process, we are developing personally, and our relationships keep us grounded and anchored. We process issues in the institutions in which we work and the feedback from agencies providing funding of our work. We are supportive through loss of loved ones and collaborators. No better example was the loss of our colleague and friend, Dr. Debra Wiegand who died in 2018 whose life was focused on building family science through relationships. Members of our group did much to see that her legacy lives on in her students, completed manuscripts, and finished analyses^(7,8,9)^. When all the other facets of the work crumble, relationships are the parts of the boat that help it steer into calm waters and stay on course and into the next port.

## Call to Action

In this paper we describe the history, context, and outcomes of our long-term collaboration as well as our recent experience as Visiting Professors. We argue that relationships make research and researchers whole because they are a way to steward the profession and are the invisible strings that connect us to one another. We provide tips for tips for growing impactful international research collaborations. Certainly, our own collaborations have resulted in almost ten presentations and five peer reviewed articles, other training opportunities, and fellowships^(6,10,11,12,13)^. We invite you to remember these lessons learned in your own professional lives as they will benefit not only yourselves and the institutions that support us but also our wider communities, our research participants and partners, students, and collaborators.
